# Physical Injuries and Hair Corticosterone Concentration in Rabbit Kits from Single- and Group-Housed Does Kept on a Commercial Farm

**DOI:** 10.3390/ani13020196

**Published:** 2023-01-04

**Authors:** Dana Hube, Joana Bill, Eric Samuel Knop, Swetlana Herbrandt, Nicole Kemper, Michaela Fels

**Affiliations:** 1 Institute for Animal Hygiene, Animal Welfare and Farm Animal Behaviour, University of Veterinary Medicine Hannover, Foundation, Bischofsholer Damm 15, D-30173 Hannover, Germany; 2 Statistical Consulting and Analysis, Center for Higher Education, TU Dortmund University, Vogelpothsweg 78, D-44227 Dortmund, Germany

**Keywords:** fattening rabbits, kits, skin lesions, hair corticosterone, group housing

## Abstract

**Simple Summary:**

On commercial farms, adult female rabbits (does) are usually kept in single cages without direct contact with conspecifics other than their kits. However, wild rabbits live together in groups with several adults and their kits, and single housing of adult domestic rabbits may affect both, their own welfare and the welfare of their kits. In this study, we focused on the welfare of kits and compared three different housing systems for does and their kits: single housing of does with their respective kits; pairs of does with their kits; and groups of three does with their kits. The kits’ health and stress level were analyzed. The number of kits with skin lesions increased with increasing group size. However, when does were pair-housed, kits seemed to have fewer disease symptoms compared to the other treatments. With regard to the stress level, there was no clear result; however, kits in groups of three does did tend to have higher stress hormones in hair. We conclude that pair housing for does could be an alternative to single housing when considering the welfare of their kits. Further studies are needed to evaluate both, the effects of pair housing on kits and does.

**Abstract:**

In intense breeding programs, rabbits are exposed to numerous stress factors which could affect their welfare and health. It has been suggested that group housing is more comfortable for does and similar to the living conditions of European wild rabbits. In this study, we compared three different housing systems—single housing; housing in pairs; and housing in groups of three does—to test whether there is a measurable impact on skin lesions, health, and hair corticosterone concentration (HCC) of their kits. The number of kits with lesions increased with the number of does kept together. The probability of kits getting injured was higher in groups of three does than in pens of single-housed does (*p* = 0.041). When does were pair-housed, kits seemed to have fewer disease symptoms compared to the other treatments. Concerning HCC of kits, there was no significant difference between the housing systems (*p* > 0.05). The mean HCC of kits was 2.94 pg/mg, while pair housing had the lowest HCC (2.59 pg/mg). This study focused on the welfare of kits from group-housed does. From this perspective, pair housing of does may be appropriate to allow social interaction between does without unduly affecting the welfare of their kits.

## 1. Introduction

Although rabbits live in social groups in the wild, adult domestic rabbits are commonly individually housed in intensive farming systems, even though it is known that single-housed rabbits often perform abnormal behaviors (digging, floor chewing, bar biting), which is an indicator of compromised welfare [1,2,3]. The gregarious nature of wild rabbits suggests that group housing is more comfortable for domestic rabbits than single housing, possibly increasing animal welfare [4]. Consequently, rabbit does would benefit from social companionship [5], even if group housing for does might entail other problems, such as physical injuries as a result of aggressiveness, territoriality, and resource guarding [5,6,7], as well as disease transmission [1]. Additionally, higher mortality of kits can occur as a result of multiple kindling in the same nest box [8] or due to undesired behavior of does towards kits, such as scraping them out of their nests [4]. However, it has to be considered that mortality is also influenced by other factors, such as parity of does [9].

Whereas, in the past, research was usually focused on does, there is still insufficient knowledge regarding rabbit kits. Some researchers have already found an influence of group housing on the does’ offspring, with a change in the number of nest box visits and a change in suckling behavior being reported [10,11], which could have a strong impact on kits. Again, Mykytowycz and Dudzinski [12] observed that does in group housing tended to be aggressive towards kits from other litters, with a strong tendency for aggressiveness to increase with advancing age of the kits, including, at the time of weaning, against their own litter, as well. Furthermore, aggressive interactions between group-housed does may increase the risk of injury to their kits. Buijs and Tuyttens [13] found that kits of semi-group-housed mothers covered shorter distances and took longer to leave the start corner in an open field test compared to kits of single-housed does. Furthermore, longer anogenital distances were measured in newborn kits from does in semi-group housing compared to single housing [14], possibly indicating that stress associated with housing conditions of does might be a prenatal influencing factor causing physical changes in kits. Whether rabbit kits from group-housed does experience stress and to what extent they sustain injuries has not been investigated to date. While the number and severity of injuries can be easily determined, measuring stress levels in small animals is more challenging. An exposure to stressors is associated with an increased hypothalamic–pituitary–adrenal (HPA) axis activity, and therefore, the response of glucocorticoids (GCs), as its end product, is considered an indicator of stress [15].

GC analysis in mammals is possible in blood, saliva, urine, feces, milk, and hair [16], as well as in liver and gonadal tissues [17]. However, the GC concentration in blood, saliva, urine, feces, and milk represents only a retrospective timespan of a few minutes up to one or two days [15]. The GC concentration can also be biased by acute stress during animal capture and handling [18]. The advantage of GC measurement in hair is that it indicates an average GC concentration over a longer period [19], depending on hair length and growth rate [15]. It has an easy and minimally invasive sampling procedure, is painless, and shaving and sampling do not require the presence of a professional [15]. A relatively small quantity of hair is required for analysis (≤500 mg) [19], and it has high long-term stability over months and years [15,20]. Consequently, hair is much easier to work with logistically.

Hair GC concentration analysis has been suggested as a non-invasive method to monitor rabbit well-being [21,22]. Cortisol is the principal GC in most mammals, like in hares [18], and corticosterone is the major GC in rodents [15]. According to Szeto et al. [23], in rabbits, cortisol and corticosterone respond similarly to social stress, and are good indexes of the stress response [3]. In an earlier study, multiple measurements of the same rabbit hair samples resulted in lower coefficients of variation for corticosterone than for cortisol. Therefore, corticosterone was recommended for hair analyses in domestic rabbits [24].

The aim of the present study was to elucidate whether the housing system of does (single housing, in pairs, or groups of three does) on a commercial rabbit farm had a measurable impact on the welfare of their kits. Therefore, the occurrence of skin lesions and the hair corticosterone concentration of the kits were determined and compared between the different housing systems.

## 2. Materials and Methods

### 2.1. Animals, Housing, and Handling

The present study was carried out on a commercial rabbit farm in Lower Saxony, Germany, where approximately 600 rabbits for breeding purposes (HYPLUS PS 19) and their offspring (HYPLUS PS 19x PS 59, HYPHARM S.A.S., Sèvremoine, France) were kept in a semi-intensive reproduction system with a 42 d reproductive rhythm and artificial insemination at day 11 postpartum (pp).

**Figure 1:**
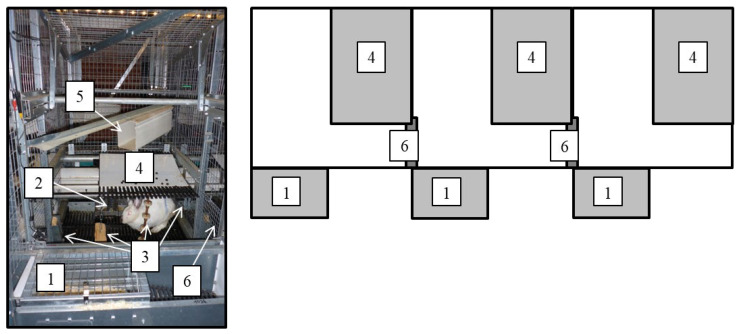
Photograph of one individual unit and scheme of a group pen for three does with nests (1), feeders (2), enrichment (3), platforms (4), plastic tube (5), and doors (6) for joining individual units to a group pen (adapted with permission from Bill et al. [25]. 2020, Elsevier).

For this study, a housing system for does with 36 individual units was used. These individual units could be joined together to form group pens for a variable number of does. For this purpose, the units were connected by doors (25 cm × 22 cm). The size of each individual unit was about 80 cm × 80 cm. The units had wire mesh walls and were open at the top. The plastic slatted floor of each unit had 11-mm-wide slots and slats. On the back wall of each unit, an elevated platform (58 cm × 52 cm) was installed. The elevated platform was not fully slatted, since part of it (40 cm × 52 cm) was covered by a plastic mat. The platform was inclined by 9% to drain off urine. Environmental enrichment was offered in each individual unit: a plastic tube (placed above the platform), a chain hanging from the platform, and gnawing material (a piece of wood attached to a chain hanging from the platform, and a second piece of wood as well as a cotton rope, both attached to the wall). The morning after the does had entered the housing system, nest boxes (25 cm × 39 cm × 32 cm) attached to the outside of each individual unit and filled with wood shavings were opened up for nest building. Figure 1 depicts the housing system, which has already been described by Bill et al. [25]. The animals were fed a diet for lactating does (Fok Lapin, Victoria Mengvoeders B.V., Veghel, The Netherlands) ad libitum; additionally chopped hay was offered. Furthermore, each unit was equipped with two nipple drinkers whereby water was constantly available. Manure was collected in a slurry store. During the study period, the mean temperature in the vacuum ventilated building was 19.5 ± 2.4 °C, and the mean humidity was 77.8 ± 9.8%. The housing system was lit by LED light strips that were switched on from 05:30 h to 19:00 h with dimming sequences of half an hour each. In addition, there were windows allowing daylight to enter the stable [25].

Does were moved to three different housing treatments (single housing, in pairs or groups of three does) one week before parturition. Two to three days before kindling, doors between single units were closed and all does were single-housed. After 18 days, the doors were opened again and the does returned to their groups, now together with their kits (postpartal mixing). Until the day of insemination, controlled suckling took place once a day by opening the nest boxes manually. Afterwards, nest boxes were constantly open to enable free suckling. At day 21 pp, all nest boxes were closed to fortify kits to eat solid food [25]. In total, 60 kits were selected for the study, 20 per type of housing (Table 1). Half of the kits were male, the other half female.

### 2.2. Lesion Scoring and Health Status

At day 30 pp, i.e., the day of weaning, all kits were weighed individually and their health status was scored as either 0 = adspectorally healthy or 1 = with disease symptoms (additionally all symptoms were recorded separately). The occurrence of injuries was scored for each individual kit according to the following scheme: 0 = no injuries; 1 = slightly and superficially injured; 2 = deep injuries; and 3 = partial loss of body parts (e.g., ears).

### 2.3. Hair Samples

At day 30 pp, hair samples for corticosterone measurement were collected. Using commercially available electric clippers for small animals, a symmetric area of 2 cm × 2 cm was shaved laterally on the hind leg as close as possible to the skin. The hair was stored in airtight plastic bags under light-protected and dry conditions at room temperature to avoid a possible washout effect by UV radiation [15]. The hair samples were sent to the Dresden LabService GmbH, Dresden, Germany for analysis. One sample per kit was collected and analyzed.

### 2.4. Measurement of Hair Corticosterone Concentration

#### 2.4.1. Corticosterone Extraction

The impact of contamination by sweat, sebum, or excreta may be minimal if samples are washed [15,19,20]. The hair washing and steroid extraction procedures were based on the protocol described in Stalder et al. [26] (study II) with modifications made by Gao et al. [27] to allow analysis by liquid chromatography coupled with tandem mass spectrometry (LC–MS/MS): Hair strands were washed by shaking them in 2.5 mL isopropanol for 3 min at room temperature. They were then allowed to dry under a fume hood for at least 12 h. A total of 10 mg of whole, non-pulverized hair was carefully weighed and transferred into a 2 mL tube (Eppendorf AG, Hamburg, Germany). After this, 50 μL internal standard and 1.8 mL methanol were added, and the hair was incubated for 18 h at room temperature for steroid extraction. Samples were spun in a centrifuge at 10,000 rpm for 2 min and 1 mL of the clear supernatant was transferred into a new 2 mL tube. The alcohol was evaporated at 65 °C under a constant stream of nitrogen until the samples were completely dried (duration: approximately 20 min). The dry residue was re-suspended using 250 μL distilled water, 200 μL of which was used for LC–MS/MS analysis [27].

#### 2.4.2. Corticosterone Concentration by LC-MS/MS

The HCC was analyzed using LC–MS/MS, which eliminates cross-reactivity, since each steroid has a unique molecular mass-to-charge ratio, thus ensuring high specificity [28]. The lower detection limit of this protocol is 0.3 pg/mg for corticosterone. All samples were analyzed in one charge. The intra-assay coefficient of variation (variation within plates) of this assay was below 9.3%.

### 2.5. Statistical Analysis

Statistical analyses were performed using the statistical software R [29]. The level of significance was set at *p* < 0.05. Data were checked for normality using histograms. The measured corticosterone values were logarithmized to approach a normal distribution. Since no kits had a lesion score of 2 and only two kits a score of 3, lesion scores of 1 to 3 were combined for the statistical models, resulting in a binary lesion variable, with the feature values “yes” = any type of lesions, or “no” = no lesions.

#### 2.5.1. Lesions

To test whether the group size of the does had an impact on the lesions of the kits, at first, a logistic regression model [30] was used with lesion as dependent variable and group size as independent variable. The Akaike information criterion (AIC) [30] was calculated to assess the model quality. Afterwards, further independent variables like sex, weight, health status, and HCC were added individually to the model to see whether they decreased the AIC of the model. Finally, an F-test was conducted for the model with the lowest AIC, with group size and weight as independent variables to test which of these independent variables had a significant impact on the lesion variable. In case of significance, pairwise comparisons using Tukey’s method [31] were additionally conducted using the R package “emmeans” [32].

#### 2.5.2. Hair Corticosterone Concentration

To test whether different factors had an impact on the HCC of the kits, a linear regression model [30] was used with logarithmized HCC as the dependent variable and group size, sex, health status, and lesion as independent variables. Again, an F-test was conducted. Since no significant results were obvious in the F-test, no pairwise comparisons were carried out.

## 3. Results

### 3.1. Body Weight, Lesions, and Health Status

The mean body weight of all kits at weaning was 765.15 g ± 123.08 g. Kits from groups of three does had the lowest body weight at weaning, with a mean of 711.55 g ± 116.44 g, followed by kits of single-housed does (729.35 g ± 112.55 g). Kits of pair-housed does had the highest mean body weight at weaning (854.55 g ± 84.10 g). A total of 19 of 60 kits (five males and 14 females) had disease symptoms, with the majority of kits suffering from rhinitis (13 kits, i.e., 68.4% of the diseased kits). Other disease symptoms of kits were conjunctivitis in one or both eyes, mild pollution of the genitals, and central nervous symptoms. Kits of pair-housed does had the lowest number of symptoms (20%), followed by kits from groups of three does (35%), and kits from single-housed does (40%). A total of 29 kits (12 males and 17 females) had skin lesions. The number of kits with lesions increased with the number of does per group, and therefore also with the number of kits kept together (kits from single-housed does = 25%, kits from pair-housed does = 55%, kits from groups of three does = 65%).

The results of the logistic regression model are shown in Table 2, Table 3 and Table 4. The results of the F-test showed significant differences regarding the risk of injury between at least two group sizes (*p* = 0.026), but not for body weight (*p* > 0.05) (Table 3). Compared to kits from single-housed does, the odds of getting injured increased when kits were kept in cages with pair-housed does (*p* = 0.056) and they increased significantly when kits were raised in cages housing three does (*p* = 0.041). There was no significant difference between the housing treatments pairs and groups of three (*p* > 0.05) (Table 4).

### 3.2. Hair Corticosterone Concentration

**Figure 2:**
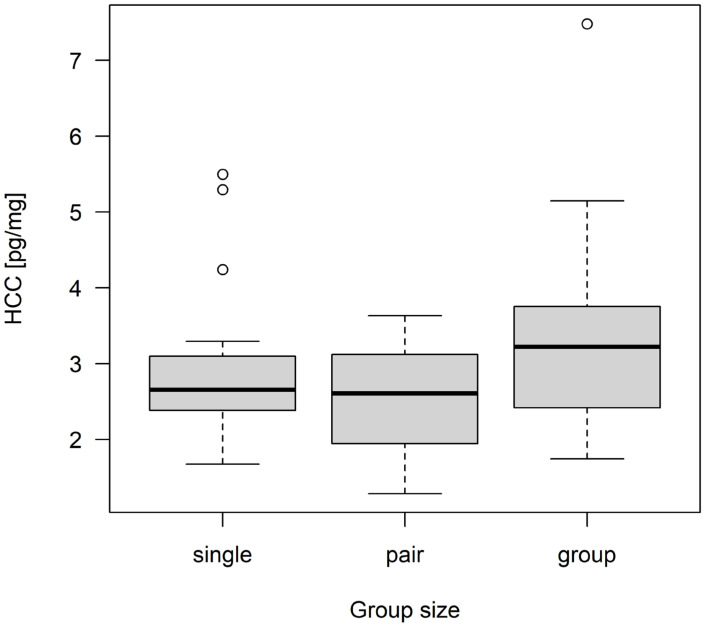
Hair corticosterone concentration (HCC) of kits in different housing treatments (single housing, in pairs or groups of three does). The HCC values [pg/mg] are shown as box plots with minimum, lower quantile, median, upper quantile, maximum, and outliers (N = 60 kits).

**Figure 3:**
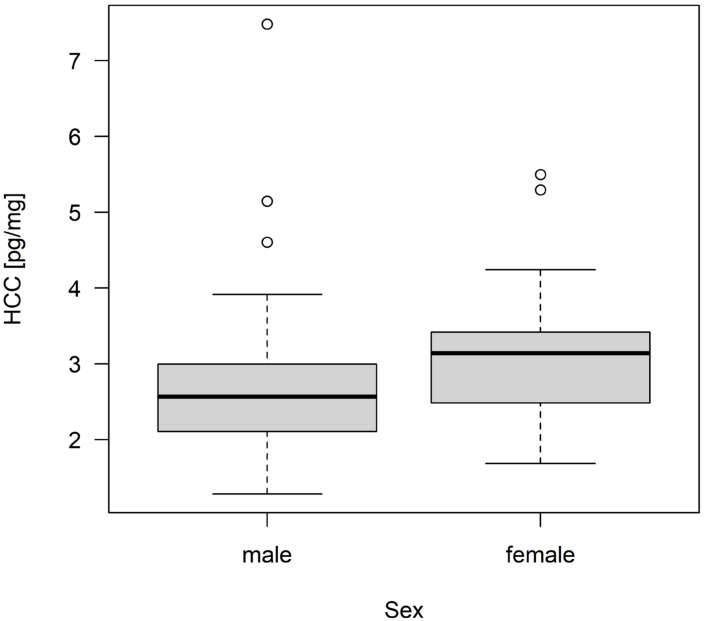
Hair corticosterone concentration (HCC) of male and female kits. The HCC values [pg/mg] are shown as box plots with minimum, lower quantile, median, upper quantile, maximum, and outliers (N = 60 kits).

The mean HCC of all kits was 2.94 pg/mg ± 1.06 pg/mg (minimum: 1.29 pg/mg; maximum: 7.48 pg/mg). The results of the linear regression model are shown in Table 5 and Table 6. There was no significant impact of group size, sex, health status, and lesions on the HCC of kits (Table 6). However, it was descriptively shown that kits originating from groups of three does tended to have higher HCC values than kits from single-housed and pair-housed does (Figure 2). This was also reflected in the estimates (Table 5). Kits of pair-housed does had the lowest HCC, with a mean of 2.59 pg/mg, followed by kits of single-housed does with a mean of 2.91 pg/mg. The highest mean HCC was found for kits originating from groups of three does (3.34 pg/mg). Female kits had slightly higher HCC values than male kits (Figure 3).

## 4. Discussion

In this pilot study, HCC of rabbit kits were presented as a possible new marker for the welfare of young animals from birth to weaning. Based on HCC and skin lesions of kits, three different housing systems of does were compared on a commercial farm: single housing, pairs of does, or groups of three does. The aim of the study was to assess these housing systems regarding their effects on rabbit kits. In the following, the results of the study are discussed in detail.

### 4.1. Lesions

As the group size of does increased, the occurrence of lesions in their kits also increased. For rabbits, fights were not observed before 12 weeks of age [5]. For females, Whary et al. [33] suggested that their immaturity is one reason for that. Additionally, food competition should not have been a problem here [1], since there was a sufficient amount of food available and kits were still suckling. Consequently, it is more likely that cage furniture is a potential risk factor for injuries [33]. Nonetheless, in pair and group housing, a much more likely reason is does inflicting injuries on kits. Mykytowycz and Dudzinski [12] observed that does tended to be aggressive towards kits from litters other than their own, with a strong tendency for aggressiveness to increase with the age of kits. In the wild, this rarely occurs, since a series of territorial arrangements protects the kits from being attacked by unfamiliar does [12]. According to Buijs et al. [7], does are also more aggressive in close proximity to their nests. Thus, due to space limitations in fattening cages, the likelihood of social stress and lesions may increase. On the contrary, Rommers et al. [10] observed that in group housing, does often suckled a mix of their own and other kits. However, at the age of 22–30 days pp, when the kits are weaned and the time of birth of the next litter is approaching, the does also become more aggressive towards their own kits [12]. In future studies, it would also be interesting to investigate whether unfamiliar kits are fully accepted by the does after cross-fostering or whether the does are more aggressive towards them than towards their blood-related kits.

### 4.2. Hair Corticosterone Concentration

Since rabbits are born naked [34], the corticosterone concentration in the clipped hair is assumed to resample the long-term stress experienced since their birth and is comparable with the shave/re-shave method [15,20]. However, it is quite possible that the period from birth is not fully reflected when HCC is measured in the kits’ hair, since there is hardly any scientific data on the speed at which hair grows in kits. According to Perdue et al. [35], by 8 days of age, hair growth in kits is considerable. It can therefore be assumed that hair formation begins well before the 8th day. Interestingly, the occurrence of lesions or disease symptoms in this study was not reflected by the HCC of kits. One reason for this could be that the health status diagnosis is a snapshot compared to the HCC which should be an indicator for long-term stress. Consequently, the HCC value might be lower than expected for some kits because they might have been recently injured, or higher than expected because some kits might have been sick or injured and recovered by the time of the health status assessment. The mechanisms concerning the incorporation of the hormone in the hair shaft have not been fully investigated yet. Nevertheless, it is currently assumed that hair GCs reflect adrenocortical activity [36], and hair has become a favored substrate for GC concentration analysis [37]. Consequently, hair GC concentration analysis has already been used for more than 40 species [38]. However, to the best of our knowledge, it has not yet been used for rabbit kits. This gap should be closed with the present study, in which HCC values for kits are now presented.

A significant connection between the group size of the does and their kits’ HCC was not found. However, there is still the possibility that a connection exists, like the tendency for higher HCC values in groups of three does compared to those in pairs of does implies. In a prior study of our research group, there was also no clear tendency when comparing the hair cortisol concentration of kits from does housed singly or in groups of three or five [39]. Trocino et al. [40] found that fattening rabbits of a HYPLUS crossbred line showed higher HCC values in collective pens (20–54 rabbits/pen) compared to pair housing. Nonetheless, these findings might not be applicable to our study due to the large gap between the group sizes and the age of the rabbits. Again, in other mammals, namely sheep and meerkats, the salivary and fecal GC concentrations decreased with increasing group size [41,42].

A significant influence of sex on HCC was also not seen in our study. Cabezas et al. [43] found no significant differences in serum corticosterone and fecal GC metabolite levels enhanced by stress between male and female wild rabbits, either. On the other hand, Monclús et al. [44] observed that male wild rabbits experienced a higher increase in corticosterone metabolites in feces than females due to fox odor. It is possible that the GC response of males and females depends on the type of stressor and the age of the rabbits. Considering other species where the hair GC concentration was measured, in polar bears [17,45] and black bears [46], as well as in non-human primates [47], an influence of sex was found, but not in wild house mice [48], wild white-footed mice [49], rats [50], and in grizzly bears [51]. However, it must be taken into account that the animals in all these studies were already sexually mature, which could also be an explanation for why there was no difference between male and female kits in our study. For example, Waterhouse et al. [52] stated that higher corticosterone levels in female pikas compared to male ones most likely reflected the physiological demands of reproduction.

There also might be other reasons for increased HCC values than those of group size of the does and the sex of the kits. Janicki et al. [53] suggested that cage breeding induces a certain amount of constant stress for adult brown hares, which is; however, insufficient to be considered a significant influence on reproduction efficiency and health status. Morgan and Tromborg [54] listed many other stressors for animals in captivity that can lead to increased GC production. Consequences can be, for example, increased aggressiveness and self-injury [54]. For snowshoe hares, maternal stress during parturition also alters HPA activity and GC levels in offspring, leading to reduced growth and viability of the kits [55]. These effects are also multigenerational, with persistent increases in HPA activity and reduced fecundity also affecting the litters of offspring born to stressed females [45,56]. This inheritance of stress could also be a reason caged rabbits may be permanently stressed, which may have masked the effects of the different housing types on HCC in our study. The reasons for stress in does should therefore also be investigated and minimized.

## 5. Conclusions

In this pilot study, the effects of keeping does in groups were investigated; however the focus was not on the does, as has been usual in previous studies, but on the welfare of their kits. Furthermore, HCC values in rabbit kits were presented for the first time. It was shown that the treatment “group housing of three does” had the most kits with lesions and high HCC values. Statistically significant results were only found for lesions. In contrast, it was also shown that pair housing of does did not seem to be disadvantageous compared to single housing of does when considering welfare indicators for kits. From this perspective, a group size of two does seems to have the potential to facilitate social interaction between does without unduly affecting the welfare of the kits. However, this should be considered a preliminary result due to the limited sample size of this study.

In view of this pilot study, further research should be carried out that takes into account not only the welfare of does but also that of their kits in order to evaluate these initial results using a larger sample size. Furthermore, a comparison of HCC of does and their kits would be interesting for future studies.

## Figures and Tables

**Table 1:** Number and housing treatment (group size of does) of male and female kits used for the study.

Housing Treatment	Pen No.	No. of Male Kits Used for the Study	No. of Female Kits Used for the Study	Total No. of Kits per Pen
single	1	4	4	8
single	2	6	0	9
single	3	0	6	9
pair	4/5	5	5	15
pair	6/7	5	5	14
group of three	8/9/10	10	10	26

**Table 2:** Table of coefficients for the logistic regression model with lesions of kits as dependent variable.

	Estimate	Odds Ratio	Std. Error
Intercept	1.8855	6.5896	2.0312
weight	−0.0042	0.9958	0.0028
pair (single)	1.8827	6.5715	0.8197
group of three (single)	1.7323	5.6538	0.7164
group of three (pair)	−0.1504	0.8603	0.7563

**Table 3:** Results of F-test for the logistic regression model with lesions of kits as dependent variable.

Model Term	df1	df2	F-Ratio	*p*-Value
group size	2	inf	3.659	0.0258 *
weight	1	inf	2.232	0.1352

* *p* < 0.05.

**Table 4:** Results for the pairwise comparisons in the logistic regression model with lesions of kits as dependent variable.

Contrast	Estimate	Odds Ratio	SE	z-Ratio	*p*-Value
pair—single	1.88	6.57	0.820	2.297	0.0562
group of three—single	1.73	5.65	0.716	2.418	0.0413 *
group of three—pair	−0.15	0.86	0.756	−0.199	0.9784

* *p* < 0.05.

**Table 5:** Table of coefficients for the linear regression model with hair corticosterone concentration (HCC) of kits as dependent variable.

	Estimate	Std. Error
Intercept	0.9460	0.1120
female (male)	0.1265	0.0882
with symptoms (healthy)	−0.0257	0.0963
lesions (no lesions)	−0.0221	0.0900
pair (single)	−0.1033	0.1071
group of three (single)	0.1312	0.1077
group of three (pair)	0.2345	0.1024

**Table 6:** Results of F-test for the linear regression model with HCC of kits as dependent variable.

Model Term	df1	df2	F-Ratio	*p*-Value
group size	2	54	2.629	0.0814
sex	1	54	2.060	0.1570
health status	1	54	0.071	0.7908
lesion	1	54	0.060	0.8072

## Data Availability

The data presented in this study are available on reasonable request from the corresponding author. The data are not publicly available due to privacy.
